# 1,1′-(Butane-1,4-di­yl)diimidazole-3,3′-diium tetra­chloridozincate(II) dihydrate

**DOI:** 10.1107/S160053680800874X

**Published:** 2008-04-04

**Authors:** Ying-Hui Yu, Ai-E Shi, Yu Su, Guang-Feng Hou, Jin-Sheng Gao

**Affiliations:** a College of Chemistry and Materials Science, Heilongjiang University, Harbin 150080, People’s Republic of China

## Abstract

In the title compound, (C_10_H_16_N_4_)[ZnCl_4_]·2H_2_O, the cation lies abouton a center of inversion and the anion about a twofold rotation axis. The Zn^II^ atom is four-coordinate in a tetra­hedral environment. The cations, anions and water mol­ecules are linked by N—H⋯O, N—H⋯Cl and O—H⋯Cl hydrogen bonds into a two-dimensional network.

## Related literature

For background and the synthesis of 1,1′-(1,4-butanedi­yl)diimidazole, see: Ma *et al.* (2003 ▶)
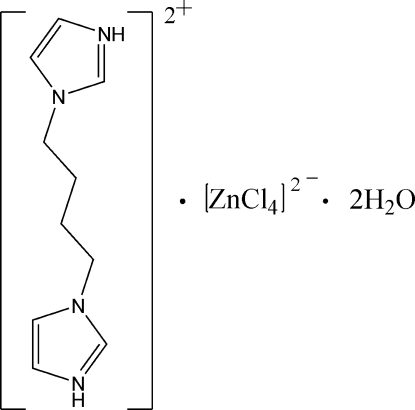


## Experimental

### 

#### Crystal data

(C_10_H_16_N_4_)[ZnCl_4_]·2H_2_O
*M* *_r_* = 435.47
Monoclinic, 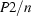

*a* = 7.4010 (15) Å
*b* = 10.927 (2) Å
*c* = 11.058 (2) Å
β = 95.23 (3)°
*V* = 890.6 (3) Å^3^
*Z* = 2
Mo *K*α radiation
μ = 1.99 mm^−1^
*T* = 291 (2) K
0.18 × 0.17 × 0.15 mm

#### Data collection

Rigaku R-AXIS RAPID diffractometer
Absorption correction: multi-scan (*ABSCOR*; Higashi, 1995 ▶) *T*_min_ = 0.713, *T*_max_ = 0.751
8575 measured reflections
2042 independent reflections
1760 reflections with *I* > 2σ(*I*)
*R*_int_ = 0.042

#### Refinement

*R*[*F* ^2^ > 2σ(*F* ^2^)] = 0.032
*wR*(*F* ^2^) = 0.081
*S* = 1.07
2042 reflections
101 parameters
H atoms treated by a mixture of independent and constrained refinement
Δρ_max_ = 0.47 e Å^−3^
Δρ_min_ = −0.37 e Å^−3^

### 

Data collection: *RAPID-AUTO* (Rigaku, 1998 ▶); cell refinement: *RAPID-AUTO*; data reduction: *CrystalStructure* (Rigaku/MSC, 2002 ▶); program(s) used to solve structure: *SHELXS97* (Sheldrick, 2008 ▶); program(s) used to refine structure: *SHELXL97* (Sheldrick, 2008 ▶); molecular graphics: *SHELXTL* (Sheldrick, 2008 ▶); software used to prepare material for publication: *SHELXL97*.

## Supplementary Material

Crystal structure: contains datablocks global, I. DOI: 10.1107/S160053680800874X/ng2438sup1.cif

Structure factors: contains datablocks I. DOI: 10.1107/S160053680800874X/ng2438Isup2.hkl

Additional supplementary materials:  crystallographic information; 3D view; checkCIF report

## Figures and Tables

**Table 1:** Hydrogen-bond geometry (Å, °)

*D*—H⋯*A*	*D*—H	H⋯*A*	*D*⋯*A*	*D*—H⋯*A*
O1—H10⋯Cl3^i^	0.85	2.43	3.275 (2)	177
O1—H9⋯Cl2^ii^	0.85	2.52	3.337 (3)	161
N2—H3⋯Cl2^i^	0.85 (3)	2.82 (3)	3.350 (2)	122 (3)
N2—H3⋯O1	0.85 (3)	2.15 (3)	2.890 (3)	145 (3)

Symmetry codes: (i) 
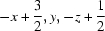
; (ii) 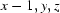.

**Fig. 1.**
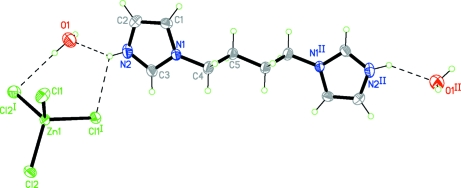
The molecular structure of (I), showing displacement ellipsoids at the 30% probability level for non-H atoms. Dashed lines indicate the intramolecular hydrogen bonding interactions.

**Fig. 2.**
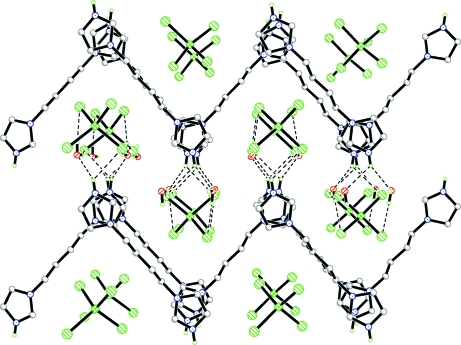
A partial packing view, showing the two-dimensional hydrogen-bonding network. Dashed lines indicate the hydrogen-bonding interactions. H atoms not involved in hydrogen bonds have been omitted for clarity.

## supplementary crystallographic information

### Comment

The 1,1'-(1,4-butanediyl)diimidazole can be used as a flexible ligand to construct coordination polymer materials(Ma *et al.*., 2003). In our attempt to synthesize the zinc complex with the 1,1'-(1,4-butanediyl)diimidazole, we unexpectedly obtained the title compound (I). Herein, we report its crystal structure.

The Zn^II^ atom lies on an inversion center and is coordinated by four chlorine anions in an tetrahedronal environment(Figure 1). The 1,1'-(1,4-butanediyl)diimidazole molecule also lies on an inversion center and its N atom is protonated.

In the crystal structure, the cations and anions are linked by N—H···Cl hydrogen bonds. In addition, the water molecules are both as acceptor and donor of hydrogen bond link these molecule into a two-dimensional supramolecular network *via* N—H···O, O—H···Cl hydrogen bonds (Table 1; Figure 2).

### Experimental

1,1'-(1,4-Butanediyl)diimidazole was prepared of imidazole and 1,4-dibromobutane in dimethylsulfoxide solution (Ma *et al.*., 2003). ZnCl_2_ (0.272 g, 2 mmol) and 1,1'-(1,4-butanediyl)diimidazole (0.380 g, 2 mmol) were dissolved in hot methanol solution (15 ml) and added two drops hydrochloric acid then a clear solution was obtained. The resulting solution was allowed to stand in a desiccator at room temperature for several days. Colroless crystals of (I) were obtained. Unexpectedly, the salt-type adducts of this ligands was crystallized from solution.

### Refinement

H atoms bound to C atoms were placed in calculated positions and treated as riding on their parent atoms, with C—H = 0.93 Å (Caromatic) and with *U*_iso_(H) = 1.2*U*_eq_(C). The N-bound H atoms were located in a difference Fourier map and free refined, Water H atoms were initially located in a difference Fourier map but they were treated as riding on their parent atoms with O—H = 0.85 Å, H···H = 1.39 and with *U*_iso_(H) = 1.5*U*_eq_(O).

### Crystal data

**Table:**

(C_10_H_16_N_4_)[ZnCl_4_]·2H_2_O	*F*_000_ = 444
*M**_r_* = 435.47	*D*_x_ = 1.624 Mg m^−^^3^
Monoclinic, *P*2/*n*	Mo *K*α radiation λ = 0.71073 Å
Hall symbol: -P 2yac	Cell parameters from 6883 reflections
*a* = 7.4010 (15) Å	θ = 3.2–27.5º
*b* = 10.927 (2) Å	µ = 1.99 mm^−^^1^
*c* = 11.058 (2) Å	*T* = 291 (2) K
β = 95.23 (3)º	Block, colorless
*V* = 890.6 (3) Å^3^	0.18 × 0.17 × 0.15 mm
*Z* = 2	

### Data collection

**Table:**

Rigaku R-AXIS RAPID diffractometer	2042 independent reflections
Radiation source: fine-focus sealed tube	1760 reflections with *I* > 2σ(*I*)
Monochromator: graphite	*R*_int_ = 0.042
*T* = 291(2) K	θ_max_ = 27.5º
ω scans	θ_min_ = 3.2º
Absorption correction: multi-scan(ABSCOR; Higashi, 1995)	*h* = −9→9
*T*_min_ = 0.713, *T*_max_ = 0.751	*k* = −14→14
8575 measured reflections	*l* = −14→14

### Refinement

**Table:**

Refinement on *F*^2^	Hydrogen site location: inferred from neighbouring sites
Least-squares matrix: full	H atoms treated by a mixture of independent and constrained refinement
*R*[*F*^2^ > 2σ(*F*^2^)] = 0.032	*w* = 1/[σ^2^(*F*_o_^2^) + (0.0301*P*)^2^ + 0.5173*P*] where *P* = (*F*_o_^2^ + 2*F*_c_^2^)/3
*wR*(*F*^2^) = 0.081	(Δ/σ)_max_ = 0.001
*S* = 1.07	Δρ_max_ = 0.47 e Å^−^^3^
2042 reflections	Δρ_min_ = −0.37 e Å^−^^3^
101 parameters	Extinction correction: SHELXL97 (Sheldrick, 2008), Fc^*^=kFc[1+0.001xFc^2^λ^3^/sin(2θ)]^-1/4^
Primary atom site location: structure-invariant direct methods	Extinction coefficient: 0.038 (3)
Secondary atom site location: difference Fourier map	

### Special details

**Table:**

Geometry. All e.s.d.'s (except the e.s.d. in the dihedral angle between two l.s. planes) are estimated using the full covariance matrix. The cell e.s.d.'s are taken into account individually in the estimation of e.s.d.'s in distances, angles and torsion angles; correlations between e.s.d.'s in cell parameters are only used when they are defined by crystal symmetry. An approximate (isotropic) treatment of cell e.s.d.'s is used for estimating e.s.d.'s involving l.s. planes.
Refinement. Refinement of *F*^2^ against ALL reflections. The weighted *R*-factor *wR* and goodness of fit *S* are based on *F*^2^, conventional *R*-factors *R* are based on *F*, with *F* set to zero for negative *F*^2^. The threshold expression of *F*^2^ > σ(*F*^2^) is used only for calculating *R*-factors(gt) *etc*. and is not relevant to the choice of reflections for refinement. *R*-factors based on *F*^2^ are statistically about twice as large as those based on *F*, and *R*- factors based on ALL data will be even larger.

### Fractional atomic coordinates and isotropic or equivalent isotropic displacement parameters (Å^2^)

**Table:**

	*x*	*y*	*z*	*U*_iso_*/*U*_eq_	
Zn1	0.7500	0.75494 (3)	0.2500	0.03710 (15)	
C1	0.4713 (3)	0.1878 (2)	0.2017 (2)	0.0405 (5)	
H1	0.4499	0.1104	0.1684	0.049*	
C2	0.4320 (4)	0.2946 (2)	0.1464 (2)	0.0469 (6)	
H2	0.3790	0.3056	0.0676	0.056*	
C3	0.5555 (4)	0.3335 (2)	0.3300 (2)	0.0448 (6)	
H4	0.6019	0.3752	0.3993	0.054*	
C4	0.6136 (3)	0.1221 (2)	0.4085 (2)	0.0450 (6)	
H5	0.6771	0.1641	0.4770	0.054*	
H6	0.6995	0.0680	0.3742	0.054*	
C5	0.4626 (3)	0.0464 (2)	0.4529 (2)	0.0376 (5)	
H7	0.3985	0.0040	0.3849	0.045*	
H8	0.3770	0.0997	0.4885	0.045*	
Cl2	0.87983 (10)	0.63431 (6)	0.11389 (5)	0.0543 (2)	
Cl3	0.96871 (9)	0.86758 (5)	0.35304 (6)	0.0529 (2)	
H3	0.475 (4)	0.461 (3)	0.216 (3)	0.066 (9)*	
N1	0.5489 (2)	0.21294 (16)	0.31649 (16)	0.0347 (4)	
N2	0.4846 (3)	0.3841 (2)	0.2278 (2)	0.0499 (5)	
O1	0.3280 (3)	0.6019 (2)	0.1132 (2)	0.0775 (7)	
H9	0.2192	0.6042	0.1319	0.116*	
H10	0.3814	0.6708	0.1191	0.116*	

### Atomic displacement parameters (Å^2^)

**Table:**

	*U*^11^	*U*^22^	*U*^33^	*U*^12^	*U*^13^	*U*^23^
Zn1	0.0465 (2)	0.0277 (2)	0.0365 (2)	0.000	0.00102 (15)	0.000
C1	0.0413 (12)	0.0380 (12)	0.0412 (12)	0.0001 (10)	−0.0009 (10)	−0.0065 (10)
C2	0.0495 (14)	0.0545 (14)	0.0359 (12)	0.0036 (12)	0.0001 (10)	0.0056 (11)
C3	0.0631 (16)	0.0326 (11)	0.0393 (12)	−0.0061 (10)	0.0085 (11)	−0.0020 (10)
C4	0.0403 (13)	0.0434 (13)	0.0499 (13)	−0.0007 (10)	−0.0029 (10)	0.0146 (11)
C5	0.0362 (11)	0.0354 (11)	0.0408 (12)	0.0017 (9)	0.0019 (9)	0.0055 (10)
Cl2	0.0739 (5)	0.0480 (4)	0.0405 (3)	0.0215 (3)	0.0029 (3)	−0.0024 (3)
Cl3	0.0563 (4)	0.0355 (3)	0.0639 (4)	−0.0094 (3)	−0.0101 (3)	0.0011 (3)
N1	0.0374 (10)	0.0297 (8)	0.0371 (9)	−0.0009 (7)	0.0030 (8)	0.0036 (7)
N2	0.0659 (15)	0.0330 (10)	0.0522 (12)	0.0040 (10)	0.0135 (10)	0.0086 (9)
O1	0.0740 (15)	0.0611 (13)	0.0940 (17)	−0.0160 (11)	−0.0113 (12)	0.0114 (12)

### Geometric parameters (Å, °)

**Table:**

Zn1—Cl3	2.2577 (8)	C3—H4	0.9300
Zn1—Cl3^i^	2.2577 (8)	C4—N1	1.470 (3)
Zn1—Cl2	2.2782 (8)	C4—C5	1.508 (3)
Zn1—Cl2^i^	2.2782 (7)	C4—H5	0.9700
C1—C2	1.337 (3)	C4—H6	0.9700
C1—N1	1.372 (3)	C5—C5^ii^	1.521 (4)
C1—H1	0.9300	C5—H7	0.9700
C2—N2	1.362 (3)	C5—H8	0.9700
C2—H2	0.9300	N2—H3	0.85 (3)
C3—N2	1.322 (3)	O1—H9	0.8500
C3—N1	1.326 (3)	O1—H10	0.8501
			
Cl3—Zn1—Cl3^i^	113.93 (4)	C5—C4—H5	109.0
Cl3—Zn1—Cl2	108.83 (3)	N1—C4—H6	109.0
Cl3^i^—Zn1—Cl2	107.95 (3)	C5—C4—H6	109.0
Cl3—Zn1—Cl2^i^	107.95 (3)	H5—C4—H6	107.8
Cl3^i^—Zn1—Cl2^i^	108.83 (3)	C4—C5—C5^ii^	110.7 (2)
Cl2—Zn1—Cl2^i^	109.29 (4)	C4—C5—H7	109.5
C2—C1—N1	107.7 (2)	C5^ii^—C5—H7	109.5
C2—C1—H1	126.2	C4—C5—H8	109.5
N1—C1—H1	126.2	C5^ii^—C5—H8	109.5
C1—C2—N2	106.7 (2)	H7—C5—H8	108.1
C1—C2—H2	126.7	C3—N1—C1	108.14 (19)
N2—C2—H2	126.7	C3—N1—C4	125.9 (2)
N2—C3—N1	108.1 (2)	C1—N1—C4	125.95 (19)
N2—C3—H4	125.9	C3—N2—C2	109.4 (2)
N1—C3—H4	125.9	C3—N2—H3	125 (2)
N1—C4—C5	113.02 (19)	C2—N2—H3	126 (2)
N1—C4—H5	109.0	H9—O1—H10	113.5

Symmetry codes: (i) −*x*+3/2, *y*, −*z*+1/2; (ii) −*x*+1, −*y*, −*z*+1.

### Hydrogen-bond geometry (Å, °)

**Table:**

*D*—H···*A*	*D*—H	H···*A*	*D*···*A*	*D*—H···*A*
O1—H10···Cl3^i^	0.85	2.43	3.275 (2)	177
O1—H9···Cl2^iii^	0.85	2.52	3.337 (3)	161
N2—H3···Cl2^i^	0.85 (3)	2.82 (3)	3.350 (2)	122 (3)
N2—H3···O1	0.85 (3)	2.15 (3)	2.890 (3)	145 (3)

Symmetry codes: (i) −*x*+3/2, *y*, −*z*+1/2; (iii) *x*−1, *y*, *z*.

## References

[bb1] Higashi, T. (1995). *ABSCOR* Rigaku Corporation, Tokyo, Japan.

[bb2] Ma, J.-F., Yang, J., Zheng, G.-L. & Liu, J.-F. (2003). *Inorg. Chem.* **42**, 7531–7534. 10.1021/ic034846q 14606848

[bb3] Rigaku (1998). *RAPID-AUTO* Rigaku Corporation, Tokyo, Japan.

[bb4] Rigaku/MSC (2002). *CrystalStructure* Rigaku/MSC Inc., The Woodlands, Texas, USA.

[bb5] Sheldrick, G. M. (2008). *Acta Cryst.* A**64**, 112–122. 10.1107/S0108767307043930 18156677

